# NanoMLST: A High‐Throughput Bacterial Multi‐Locus Sequence Typing Workflow Using Oxford Nanopore Next‐Generation Sequencing for ESKAPE + E Pathogens

**DOI:** 10.1002/mbo3.70204

**Published:** 2025-12-18

**Authors:** Isabel García‐Pérez, Fernando Lázaro‐Perona, Diana Soledad Reyes‐Zuñagua, Jared Sotelo, María Rodríguez‐Tejedor, Javier E. Cañada‐García, Iván Bloise, Sergio Martin Portugués‐Rodríguez, Jesús Mingorance, Jesús Oteo‐Iglesias, Elias Dahdouh

**Affiliations:** ^1^ Clinical Microbiology and Parasitology Department Hospital Universitario La Paz, Instituto de Investigación Sanitaria del Hospital Universitario La Paz (IdiPAZ) Madrid Spain; ^2^ Reference and Research Laboratory for Antibiotic Resistance and Healthcare‐Associated Infections, Centro Nacional de Microbiología, Instituto de Salud Carlos III Madrid Spain; ^3^ Centro de Investigación Biomédica en Red de Enfermedades Infecciosas (CIBERINFEC), Instituto de Salud Carlos III Madrid Spain

**Keywords:** ESKAPE + E pathogens, high‐throughput Multi‐Locus Sequence Typing, Oxford Nanopore Technologies

## Abstract

Multi‐Locus Sequence Typing (MLST) is a key method for allocation of Sequence Types (STs) for bacterial isolates. Traditionally, this is performed by the Sanger sequencing method, which can be highly time‐consuming and laborious. In this study, we present NanoMLST, a high‐throughput MLST workflow using multiplex PCR, Oxford Nanopore Technologies Next‐Generation Sequencing, and the Krocus program for typing ESKAPE + E pathogens (*Enterococcus faecium* [*E. faecium*], *Staphylococcus aureus, Klebsiella pneumoniae* [*K. pneumoniae*], *Acinetobacter baumannii, Pseudomonas aeruginosa, Enterobacter* spp., and *Escherichia coli*). Bacterial isolates were obtained from the Hospital Universitario La Paz's Microbiology Department and the Centro Nacional de Microbiología. Primers that can be multiplexed in a single PCR reaction were designed for the seven housekeeping genes for each species. DNA was extracted from single colonies by heating at 95°C for 10 min, mechanical lysis at 4.20 m/s for 2 min, and then by the MagCore extraction system. Multiplex PCRs were then performed with the respective primer mixes for each species, and libraries were prepared for sequencing by ONT Flongle cells. The Krocus program was then used to determine the STs from the raw FastQ reads. STs for 221 isolates were obtained through this workflow with an average time of 12 h per 24 isolates. In line with local data, the *K. pneumoniae* and *E. faecium* isolates were relatively oligoclonal, while the rest were polyclonal. STs from representative isolates showed 100% concordance between Sanger sequencing and the proposed workflow. NanoMLST offers a fast, cheaper, and less labor‐intensive alternative for large‐scale MLST applications targeting clinically important pathogens.

## Graphical abstract

NanoMLST is a workflow suggested as a viable alternative to high‐throughput ST determination for ESKAPE + E pathogens using ONT next‐generation sequencing.

## 1 Introduction

Multi‐Locus Sequence Typing (MLST), introduced in 1998, has become a global and effective technique for characterizing bacterial isolates at a molecular level (Maiden et al. 1998). Despite great advances in Next‐Generation Sequencing (NGS), this method is still widely used due to its ability to screen large sets of isolates, lower cost per isolate compared to NGS, and discriminatory power for identifying Sequence Types (STs). It can also be used to determine the most prevalent STs for the subsequent selection of representative isolates for more precise typing using NGS techniques (such as core‐genome MLST (cg‐MLST) or Whole‐Genome Sequencing (WGS)) (Boers et al. 2012). MLST characterization is based on variations in the DNA sequence of seven housekeeping genes. Each unique combination of the alleles of these genes results in a specific ST. The study of these variations is traditionally performed through sequencing with the dideoxy chain termination method (Sanger Method; hereafter referred to as SM). However, generating results through this method is very time‐consuming and labor‐intensive (Boers et al. 2012). Using SM, each sample requires one PCR per gene, and two cycle sequencing reactions per PCR product (one for the forward strand and another for the reverse strand). This results in 21 reactions per sample, which will further require a purification step for the 14 sequencing reactions (Maiden 2006). Though this might be feasible when handling a small number of samples, it can be difficult to implement in studies where hundreds of bacterial isolates need to be characterized. In a hospital setting, this characterization can be especially important to detect high‐risk clones of multi‐drug resistant (MDR) bacteria, including the clinically important ESKAPE pathogens (*Enterococcus faecium* [*E. faecium*], *Staphylococcus aureus* [*S. aureus*], *Klebsiella pneumoniae* [*K. pneumoniae*], *Acinetobacter baumannii* [*A. baumannii*], *Pseudomonas aeruginosa* [*P. aeruginosa*], *Enterobacter* spp.) (WHO Bacterial Priority Pathogens List 2024), and *Escherichia coli* [*E. coli*], another major hospital pathogen (Raoofi et al. 2023) (these pathogens will hereafter be referred to as ESKAPE + E). Quick identification of high‐risk clones through a precise method can, in turn, be essential for establishing personalized treatments and early implementation of infection control measures.

Advances in NGS techniques, such as those developed by Oxford Nanopore Technologies (ONT), might offer a rapid solution for high‐throughput MLST screening. When properly implemented, they can provide a simple and affordable alternative to conventional MLST techniques, with high turnaround times and an accuracy above 99% (Nanopore Store n.d.; Oxford Nanopore Technologies n.d.). This could also provide a faster alternative than cg‐MLST and WGS since a much larger set of isolates can have their STs determined in a given time frame due to the relatively smaller number of reads required for MLST, resulting in being able to sequence a much higher number of isolates in a single sequencing run. As compared to other NGS technologies, ONT carries the advantage of being able to sequence long reads (over 1.5 Kb) and thereby ensuring that the entire allelic region of the housekeeping genes is covered. Moreover, sequencing through ONT generally results in a lower cost per sample (Nanopore Store n.d.; Oxford Nanopore Technologies n.d.).

Several studies presented MLST workflows using NGS techniques for different microorganisms. These include *Legionella pneumophila*, *S. aureus*, *P. aeruginosa*, and *Streptococcus pneumoniae* (Boers et al. 2012), *Mycoplasma ovipneumoniae* (Framst et al. 2024), *Cryptococcus neoformans* (Chen et al. 2015), *Trichomonas vaginalis* (Squire et al. 2020), *Listeria monocytogenes* (Takahashi et al. 2017), and *Salmonella serovars* (Singh et al. 2013). However, most of these studies rely on no longer available technologies, such as the 454 pyrosequencer, and do not include a significant set of clinically important pathogens. In this study, we present a high‐throughput protocol for MLST for the ESKAPE + E pathogens using ONT NGS with a streamlined data analysis pipeline that is easy to implement. The workflow presented in this study will hereafter be referred to as NanoMLST.

## 2 Material and Methods

### 2.1 Bacterial Isolates

The clinical isolates included in this study were obtained from the bacterial collection of the Microbiology and Parasitology Department of the Hospital Universitario La Paz (HULP), which receives bacterial isolates from the larger northern Madrid (Spain) area, and the National Center for Microbiology (CNM), which receives bacterial isolates from all over Spain. They included *E. faecium* (*n* = 32), *S. aureus* (*n* = 32), *K. pneumoniae* (*n* = 33), *A. baumannii* (*n* = 31), *P. aeruginosa* (*n* = 29), *Enterobacter cloacae* complex (*n* = 28), and *E. coli* (*n* = 36). No clinical data were used, nor was any clinical sample specifically requested for this study. Rather, the already stored organisms were reused in this study.

### 2.2 DNA Extraction

Genomic DNA was purified from bacteria grown on sheep blood agar at 37°C overnight. A single colony was suspended in 1 mL of phosphate‐buffered saline (0.01 M, pH = 7.4) and heated at 95°C for 10 min. The suspension was then lysed at 4.20 m/s for 2 min using Bead Mill Max (VWR International, USA) and 0.1 mm glass beads. After a 5‐min centrifugation at 8000*g*, 400 µL of the supernatant was used for automatic DNA extraction with a MagCore system and the MagCore Nucleic Acid Extraction Kit (Code 202; RBCBioscience, USA). The extracted DNA was eluted in a volume of 100 µL, and 5 µL of the eluted DNA was mixed with 195 µL of the working solution of the dsDNA BR Assay Kit (Q33265, Thermo Fisher Scientific, USA). The mixture was then quantified by fluorometry using the Qubit 4 fluorometer (Thermo Fisher Scientific, USA). The standards provided by this same kit were used to calibrate the instrument before each time the DNA concentrations were determined. The range of DNA concentrations obtained was between 2.1 and 64 ng/µL (median = 8.9 ng/µL). In order to avoid the inhibition of the PCR reactions, concentrations that were higher than 30 ng/µL were diluted using molecular‐grade H_2_O to a concentration of 10 ng/µL, making the range of concentrations included in the downstream PCR reaction from 2.1 to 30 ng/µL. The extracted DNA was stored at −20°C until used.

### 2.3 Primer Design and Validation

The sequences of the seven housekeeping genes of *K. pneumoniae* were obtained from the database of the Pasteur Institute (https://bigsdb.pasteur.fr/klebsiella/; last accessed on 04/09/2025), and those of *E. coli* were obtained from Enterobase (https://enterobase.warwick.ac.uk/species/ecoli/allele_st_search; last accessed on 04/09/2025). The respective genetic sequences for the rest of the species included in this study were obtained from the database of the PubMLST website (the Pasteur Scheme was used for *A. baumannii*) (Jolley et al. 2018). The sequences of the alleles were aligned using the Bioedit program (version 7.7) (Kirmani 2015), and the alignments were used as templates to determine possible zones for primer design. The strategy used for targeting the nucleotides for primer design always ensured that the amplicons result in a greater amplified area compared to SM, and always includes the area used for allele determination.

Potential primer sequences were checked for auto‐dimerization and hairpin formation using OligoCalc (http://biotools.nubic.northwestern.edu/OligoCalc.html; last accessed on 06/01/2025). Possible interactions among all the primer pairs for the seven housekeeping genes per species were checked using the multiple analyzer tool on the Thermo Fisher website (https://www.thermofisher.com/es/en/home/brands/thermo-scientific/molecular-biology/molecular-biology-learning-center/molecular-biology-resource-library/thermo-scientific-web-tools/multiple-primer-analyzer.html; last accessed on 04/09/2025). The primers (Table S1) were synthesized and purchased from Merck (Sigma Aldrich Group, Germany).

The lyophilized primers were suspended in molecular‐grade water to a concentration of 100 µM per primer (stock concentration). The individual primer pairs for each gene were then diluted 1:10 and validated using singleplex PCRs (i.e., seven different reactions per sample). The PCR reaction mix included 10 μL of 2x Phusion Master Mix and 0.6 μL of 100% DMSO (both included in the Phusion High‐Fidelity PCR Master Mix with GC Buffer, Thermo Fisher, USA), 1 μL (0.5 μM of each of the diluted forward and reverse primers), 6.4 μL of nuclease‐free water, and 2 μL of template DNA. The cycling program was set as follows: 30 s of initial denaturation at 98°C followed by 30 cycles of denaturation at 98°C for 5 s, annealing for 15 s (check Table S1 for specific annealing temperatures for the primers used per species), and extension at 72°C for 15 s; and then a final extension at 72°C for 5 min.

The PCR products (i.e., amplicons) were visualized to check for the appropriate length by agarose gel electrophoresis, as per the predicted length based on the primers' design. The seven amplicons of one isolate per species were then sequenced by SM to ensure that the correct genes were amplified by the respective primers. Briefly, 5 µL of the PCR products were cleaned using ExoCleanUp Fast (VWR, Spain), and the cycle sequencing reactions were performed using BigDYE Terminator kit v. 3.1 (Thermo Fisher, USA). The products (10 µL) were transferred into resin columns (AutoSeq G‐50 Dye Terminator Removal Kit; Cytiva, UK) and centrifuged at 800*g* for 4 min. The entire volume was then sequenced using the SeqStudio Genetic Analyzer (Thermo Fisher, USA). The forward and reverse strand sequences were visualized and aligned using the BioEdit program, and the STs were determined using the PubMLST website. In addition, equal volumes (5 µL) of the individual amplicons per species were pooled (seven amplicons per species; one isolate per species) and sequenced using ONT as described in the subsequent sections.

### 2.4 Multiplexing the PCR Reactions

All the primer pairs for each of the species were pooled into a primer mix. Table S1 shows the volumes and concentrations for each individual primer pair. The volumes for some of the primer pairs were adjusted after the initial trials that showed unequal numbers of reads for each of the seven genes. For example, one gene would be represented by 50% of the reads, another by 10% of the reads, and the remaining 40% of the reads would be distributed among the other five genes. This was corrected by lowering the concentration of the primer pairs that resulted in over‐representing genes, while increasing the concentration for the primer pairs that resulted in under‐representing genes. On average, two trials per species (range = 0–3) were needed to optimize the volumes of the primer pairs in the mix to obtain a homogeneous distribution of the number of sequences obtained per gene.

The multiplex PCRs were performed using the Phusion High‐Fidelity PCR Master Mix with GC Buffer (Thermo Fisher Scientific, Waltham, MA, United States) as described in the previous section. For each reaction, 1 μL of the primer mix was added. The thermal cycling conditions were the same as described before, and the annealing temperatures for the primer mixes are listed in Table S1. For the *A. baumannii* and *E. faecium* isolates, the extracted DNA was heated at 95°C for 10 min, and the PCR reaction mix was kept on ice until the thermocycler reached the initial denaturation temperature (95°C) before placing it in the thermal cycler in order to inactivate the nucleases. After each multiplex PCR reaction, the amplicons were visualized by gel electrophoresis.

### 2.5 Library Preparation and Sequencing

Libraries were prepared using the Nanopore Native Barcoding Kit SQK‐NBD114.24 (Oxford Nanopore Technologies, UK), according to the manufacturer's instructions. The libraries were loaded onto R10.4.1 Flongle cells (FLO‐FLG114) and sequenced using MinION Mk1C (Oxford Nanopore Technologies, UK). A minimum quality score of 8 was selected, and fast basecalling was performed using the built‐in Guppy basecaller. A trial using the high‐accuracy basecalling option was also performed for 96 samples and compared to the fast basecalling for that same run.

### 2.6 Data Analysis

Raw FastQ reads were concatenated, and the Krocus program (version 1.0.3) (Page and Keane 2018) was used to determine the STs directly from them. The ambiguities produced by Krocus (when applicable) were further investigated by mapping the reads against the reference alleles using the MLST Finder tool in the Center for Genomic Epidemiology website (http://www.genomicepidemiology.org/; last accessed on 04/09/2025). When ambiguities were discovered (marked by “?” by Krocus when a perfect hit or ST could not be found), they were resolved by manual correction using Geneious Prime (version 2025.1, Dotmatics, USA). Using this program, the reads were mapped against their respective references, consensus sequences were generated, and ambiguities were manually revised for artificial insertions and/or deletions (indels). After correction, STs were assigned using the MLST Finder tool of the Center for Genomic Epidemiology website. In order to compare the STs assigned by Krocus, 10% of the most prevalent STs per species (as determined by Krocus) had their STs determined by Geneious and MLST Finder as described above. After the determination of the STs for all the isolates, the housekeeping genes for three representative STs per species were sequenced by SM.

## 3 Results

A total of 221 (range per species = 29–36) clinical ESKAPE + E isolates were included in this study. Through NanoMLST, the coverage for all seven genes was balanced across each isolate (after the optimization steps mentioned in the methods section). The total number of reads obtained per run was highly variable and dependent on how many pores in the Flongle flow cells were active at the time of sequencing, how long the pores were active, and the total number of samples included. In all cases, the homogeneity of the coverage across the seven genes was maintained. Table 1 shows the median reads per sample, per gene, and per species.

**Table 1:** Median reads obtained per sample and per gene for each of the seven species whose sequence was determined from the first attempt through the NanoMLST workflow.

Species	Median reads per sample (range)	Median reads per gene (range)
*E. faecium*	45,313 (3509–119,980)	1384 (559–3125)
*S. aureus*	36,013 (5896–53,203)	936 (859–1853)
*K. pneumoniae*	43,735 (4121–96,717)	1060 (777–1436)
*A. baumannii*	12,567 (3782–15,838)	1284 (623–5537)
*P. aeruginosa*	141,759 (18,373–258,156)	13,427 (6072–31,990)
*Enterobacter cloacae* complex	48,214 (8000–108,000)	2174 (582–7787)
*E. coli*	19,279 (3425–36,101)	871 (626–1182)

With the described conditions, 212 isolates (95.1%) had their ST determined from the first attempt, while the remaining 11 (4.9%; excluded from Table 1) did not. Of these, there was a failure in determining an allele for three *K. pneumoniae* isolates, where, in one isolate, the gene affected was *inf*B, in another *mdh*, and in the third *pgi*. One *E. faecium* isolate had a failure in both *gyd* and *pst*S, a second in *gdh*, and a third in *pur*K. One *E. coli* isolate had a failure in *fum*C and another in *adk*. One *S. aureus* isolate had a failure in *aro*E and another in *yqil*. One last isolate of *P. aeruginosa* had a failure in *guaA*. For all these isolates, the number of reads per failed gene was below 500 reads (range: 343–495; average = 428; 95% confidence interval (CI) = 394–461) and significantly lower than the reads per genes obtained for the isolates in which STs were determined from the first run (range: 626–31,990; average = 2457; 95% CI = 1343–3570; *p* < 0.01). In addition, the total reads obtained for the former isolates (range: 1973–3851; average = 2949; 95% CI = 2526–3372) were significantly lower than those in which STs were determined from the first run (range: 3425–258,156; average = 7969; 95% CI = 7221–8717; *p* < 0.001). All the isolates that did not have their STs determined from the first run were resequenced using the same DNA extracts and had proper ST designations and read numbers within the range of those that had STs designated from the first attempt (i.e., 100% of the isolates had their STs determined). Based on this data, cutoff values of 3500 reads per isolate as an initial evaluation parameter of whether or not ST designation is likely, and another cutoff value of 500 reads per gene as a second evaluation parameter were placed.

Table 2 shows the different STs determined for the ESKAPE + E isolates. Novel STs had a combination of alleles that did not match any profile in the database used, and new STs were assigned by pubMLST (ten isolates in total).

**Table 2:** Number of isolates included in this study per species, as well as the Sequence Types (STs) identified.

Species (number of isolates)	Sequence Types (number of isolates)
*E. faecium* (32)	ST1581* (24), ST64 (2), ST695* (1), ST533* (1), ST117 (1), ST114 (1), ST27 (1), ST178 (1),
*S. aureus* (32)	ST22* (3), ST30 (2), ST45* (2), ST125 (2), ST2167 (2), ST4166 (2), ST1* (1), ST5 (1), ST6 (1), ST8 (1), ST26 (1), ST72 (1), ST88 (1), ST97 (1), ST398 (1), ST672 (1), ST938 (1), ST3706 (1), ST6058 (1), ST6089 (1), ST7650 (1), ST7672 (1) Novel STs: ST9961, ST9962, ST9963
*K. pneumoniae* (33)	ST39** (28), ST15* (5)
*A. baumannii* (31)	ST2* (15), ST79 (3), ST25 (3), ST54 (1)*, ST203 (1)*, ST1 (1), ST10 (1), ST85 (1), ST108 (1), ST238 (1), ST1528 (1), ST2585 (1) Novel ST: ST2830
*P. aeruginosa* (29)	ST253* (3), ST242* (2), ST277* (2), ST27 (1), ST175 (1), ST186 (1), ST235 (1), ST244 (1), ST270 (1), ST395 (1), ST532 (1), ST699 (1), ST1029 (1), ST1227 (1), ST2351 (1), ST3347 (1), ST3446 (1), ST4328 (1), ST4387 (1), ST4434 (1), ST4515 (1) Novel STs: ST5332, ST5333, ST5334, ST5335
*Enterobacter cloacae* complex (28)	ST45* (2), ST50* (2), ST56* (2), ST114 (2), ST145 (2), ST190 (2), ST1273 (2), ST24 (1), ST102 (1), ST113 (1), ST116 (1), ST126 (1), ST270 (1), ST423 (1), ST501 (1), ST688 (1), ST1001 (1), ST1116 (1), ST2797 (1) Novel STs: ST3325, ST3326
*E. coli* (36)	ST131* (11), ST73* (4), ST95 (3), ST405 (2)*, ST1193 (2), ST44 (1), ST69 (1), ST117 (1), ST127 (1), ST162 (1), ST404 (1), ST410 (1), ST427 (1), ST550 (1), ST636 (1), ST675 (1), ST1331 (1), ST11240 (1), ST13164 (1)

*Note:* Novel STs were assigned by the PubMLST platform. The “*” indicates that isolates pertaining to these STs were also sequenced by the Sanger Method.

Ten percent of the isolates representing the most prevalent STs per species determined by Krocus were compared to the STs determined by the MLST Finder tool of the Center for Genomic Epidemiology. Raw reads were used for Krocus, and consensus sequences were used for MLST Finder. The same STs were assigned to the respective isolates using both methods. The 21 representative isolates per species (highlighted by an asterisk “*” in Table 2) that were sequenced by SM for comparison yielded the same results for ST designation as that that were determined by NanoMLST.

Using the NanoMLST workflow, 183 isolates (82.8%) were automatically assigned STs by Krocus. The remaining 38 isolates (17.2%) had to be manually revised by Geneious. For the isolates that were also sequenced by SM, all (100%) the sequences needed to be manually curated and at least one forward or reverse sequence for one of the seven genes had to be repeated due to poor read quality, per isolate (i.e., for each isolate included in this study, there was at least one gene to be repeated, ranging from 1 to 4 genes, or from 7.1% to 28.6% of all sequences per isolate). None of the isolates sequenced by SM had their ST determined from the first run. Comparing the high‐sensitivity basecalling to the fast basecalling options for 96 isolates did not result in a significant change in the number of ambiguities generated (median of 1 ambiguity per isolate (range 0–12) using both basecalling methods). The difference between the basecalling algorithms was not significant (*p* > 0.05), and using the high‐accuracy basecalling resulted in 48 additional hours for basecalling, per run, as compared to fast basecalling.

**Figure 1:**
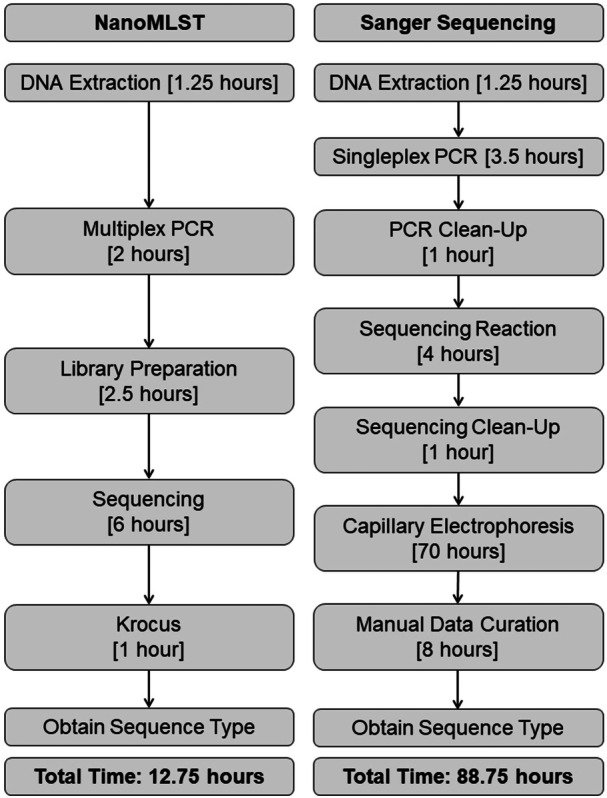
General workflow and estimated time for the completion of each step for NanoMLST compared to the Sanger sequencing method for 24 samples.

Figure 1 illustrates the steps of the NanoMLST workflow for 24 samples, as well as an estimate of the time required for each step. It also shows a comparison to the time required to perform the same for 24 samples via SM. Through NanoMLST, the time needed from DNA extraction to loading the flow cell is less than one 8‐h working day. Sequencing through MinION can start producing results within an hour, and the pores in a Flongle flow cell remain actively sequencing for, on average, 6 h. Krocus analyses on the computer that was used for this study (CPU: I7‐11700k, RAM: 64GB, SSD: 1TB, VGA: Nvidia T1000, Operating System: Ubuntu 4.1) took an average of 1 h for 24 samples. Based on this, the entire workflow can be divided between 2 working days and takes a total of 12.75 h. For the samples that required manual ST assignment using Geneious and MLST Finder (38 isolates), the average time per sample was 15 min by an experienced person (9.5 h in total). In comparison, performing SM for 24 samples requires two working days to load the samples for capillary electrophoresis. The machine will then require 3 days to process all the 336 sequences (7 sequences for the forward strand and 7 sequences for the reverse strand, per isolate). This requires several runs prepared in 96‐well plates that will be sequenced at a rate of 12 min per sequence (the rate of the machine used for this study). Afterward, an entire 8‐h working day is needed to manually analyze the sequences by an experienced person. This results in a total of 6 working days per 24 samples using SM. In terms of cost, at the time and place where this study was made, the cost per sample using NanoMLST was around 53 euros (14 of which were for plasticware), while for SM it was around 89 euros (35 of which were for plasticware).

## 4 Discussion

MLST was developed as a rapid, reproducible, and accessible approach to type pathogenic bacteria (Maiden et al. 1998). Over the years, both the methodology and the information provided have been shown to be robust, despite having higher resolution methods available (such as WGS and cg‐MLST). MinION by ONT provides a high‐throughput sequencing platform capable of generating sequences at 450 bases per second (Wang et al. 2021), allowing millions of long reads to be obtained in a relatively short time. This workflow provides a distinctive advantage over other typing methods (e.g., WGS and cg‐MLST) due to the ability to process a much larger set of isolates per run. In this study, this capacity was exploited to determine the STs of up to 24 isolates in around 12 h (equivalent to 2 working days) in a simple and fast workflow (NanoMLST). This number can be scaled up to 96 or more using other flow cells or ONT platforms, resulting in an increase in response time due to the parallel processing capability and scalability of these platforms. The determination of the STs can, in turn, provide information regarding the presence of STs associated with MDR (Girlich et al. 2015), highly virulent STs (Arcari and Carattoli 2023), and whether the same STs are spreading in different locations (Tryfinopoulou et al. 2023; Villalón et al. 2011). Moreover, multiplexing the primers for all seven housekeeping genes allows the amplification of all targets in a single PCR reaction. This has resulted in a significantly less time‐consuming and labor‐intensive workflow compared to SM, which requires a lot more hands‐one time (Figure 1). In terms of expertise, levels that are similar to those required to determine MLST by SM are needed. Namely, the experienced person will need to learn how to prepare the samples for sequencing and run the Krocus program. For those who need a manual ST designation, the person needs to learn how to map the reads, look for ambiguities (generally marked as N), compare the reads to the reference, and resolve the indels that arise. Since all the target genes are housekeeping genes, resolving indels is not especially challenging, and further expertise is not needed for the successful designation of STs by NanoMLST. Moreover, in comparison with other high‐throughput MLST typing (Boers et al. 2012; Chen et al. 2015; Squire et al. 2020; Takahashi et al. 2017; Singh et al. 2013), even those using the same NGS technology (ONT) (Framst et al. 2024), NanoMLST targets highly impactful hospital pathogens (WHO Bacterial Priority Pathogens List 2024; Raoofi et al. 2023) and a sequencing platform that is readily available in the market and fairly accessible for most laboratories. It has also been optimized to be used with relatively low‐cost, inexpensive equipment (compared with other NGS technologies) and basic levels of expertise. This makes its implementation in clinical and epidemiological settings easier than other NGS techniques and gives it the potential for widespread use.

In terms of error rates, a global percentage of 4.9% of the isolates included did not have an ST assigned to them from the first run using NanoMLST. In comparison, a range of 7.1%–28.6% of low‐quality reads using SM was detected in each of the isolates included in this study (i.e., in 100% of these isolates, at least one gene had to be repeated). Performing a sequencing run with all the isolates that required repetition by NanoMLST successfully resulted in ST assignment across all tested strains within a maximum of 2 working days, while repeating the genes with low qualities by SM required an entire week. This information, combined with the reduced cost per sample, presents NanoMLST as a viable alternative to SM for application in high‐throughput ST determination, especially since the design aims at highly important pathogens (ESKAPE + E). This fact may allow, for example, the quick determination of the prevalence of different STs in healthcare settings, a key factor in the effective control of the spread of MDR clones. Additionally, this workflow, although difficult to apply to highly complex clinical samples where several clones and species can be present (such as feces or rectal swabs), might be applicable directly to usually sterile clinical samples where monoclonal, monobacterial infections are likely (e.g., blood or cerebrospinal fluid). This can potentially reduce the time needed for obtaining isolated colonies, and is an approach that will be explored in future studies.

NanoMLST additionally allows the circumvention of the relatively high error rates of ONT (Wang et al. 2021) through a large sequencing depth. It was calculated that by including 24 samples in the Flongle flow cell, an average of 3500 reads will be obtained per sample (i.e., 500 reads per gene), allowing for a correction of ONT's error rates. However, the variability of the time pores remain active resulted in a highly variable total number of reads obtained per run, and could be the reason why 4.9% of the isolates were not assigned STs from the first run. This should be taken into consideration if the routine application of NanoMLST is to be performed, in addition to the need to apply strict criteria of the minimum number of reads acceptable for reliable results (above 500 per gene and above 3500 total reads), and ensuring that local databases are up to date.

The bacterial isolates included in this study were selected from the bacterial collections of HULP (which covers the larger northern Madrid area) and CNM (which receives isolates from all over Spain), where generally only MDR isolates are included. Therefore, the STs designated do not reflect an actual epidemiological distribution of the isolates. However, they provide a rough image of the genetic diversity found in clinical isolates of the selected species. For instance, *K. pneumoniae*, *E. faecium,* and *A. baumannii* are represented by a single major clone (ST39, ST1581, and ST15, respectively) (Tryfinopoulou et al. 2023; Villalón et al. 2011), while isolates from the rest of the organisms showed more diversity. Another important thing to note is that a large selection of STs was determined by NanoMLST (Table 2). This gives confidence in the ability of this method to detect a wide range of clones, including novel ones, and highlights its potential use in epidemiological studies and as a screening tool in the hospital setting.

Another interesting application for NanoMLST is its use to screen a collection of isolates to test for relatedness. This can be especially important since NanoMLST is able to process a large number of samples as compared to WGS (24 samples per Flongle flow cell and 96 per standard flow cell, as opposed to 1 sample per Flongle flow cell and 10 per standard cell). This enables a fast, cost‐effective typing method that can give an overview of large sets of isolates, and allows for the selection of representative ones for more in‐depth analyses. Last but not least, NanoMLST can be used for other types of large‐scale epidemiological screening of these organisms, in a manner similar to that used for avian *E. coli* (Jia et al. 2024 *)*. These applications can carry a great value in limited‐resource settings where, instead of performing WGS or cg‐MLST for all isolates in a large set, NanoMLST can be effective at determining the distribution of the STs in that set, where representative ones can be selected for higher‐resolution typing.

In conclusion, NanoMLST provides an easy‐to‐implement and rapid workflow to determine the ST of ESKAPE + E isolates and sets the ground for future large‐scale studies. These could include the possibility of adding the detection of more genes into the design, such as clinically relevant antibiotic resistance genes.

## Author Contributions

**Isabel García‐Pérez, Fernando Lázaro‐Perona, Jesús Mingorance, Jesús Oteo‐Iglesias, Elias Dahdouh:** conceptualization. **Isabel García‐Pérez, Fernando Lázaro‐Perona, Iván Bloise, Jared Sotelo, Javier E. Cañada‐García, Sergio Martin Portugués‐Rodríguez, Elias Dahdouh:** methodology. **Isabel García‐Pérez, Fernando Lázaro‐Perona, Iván Bloise, Jesús Mingorance, Jesús Oteo‐Iglesias, Elias Dahdouh:** formal analysis. **Isabel García‐Pérez, Diana Soledad Reyes‐Zuñagua, Jared Sotelo, María Rodríguez‐Tejedor, Javier E. Cañada‐García, Sergio Martin Portugués‐Rodríguez:** investigation. **Iván Bloise, Fernando Lázaro‐Perona, Elias Dahdouh:** Software. **Fernando Lázaro‐Perona, Jesús Mingorance, Jesús Oteo‐Iglesias, Elias Dahdouh:** supervision. **Fernando Lázaro‐Perona, Jesús Mingorance, Jesús Oteo‐Iglesias, Elias Dahdouh:** validation. **Fernando Lázaro‐Perona, Jesús Mingorance, Jesús Oteo‐Iglesias, Elias Dahdouh:** funding acquisition. **Isabel García‐Pérez, Diana Soledad Reyes‐Zuñagua, Jared Sotelo, María Rodríguez‐Tejedor, Javier E. Cañada‐García, Sergio Martin Portugués‐Rodríguez, Fernando Lázaro‐Perona, Iván Bloise, Jesús Mingorance, Jesús Oteo‐Iglesias, Elias Dahdouh:** writing – original draft. **Isabel García‐Pérez, Diana Soledad Reyes‐Zuñagua, Jared Sotelo, María Rodríguez‐Tejedor, Javier E. Cañada‐García, Sergio Martin Portugués‐Rodríguez, Fernando Lázaro‐Perona, Iván Bloise, Jesús Mingorance, Jesús Oteo‐Iglesias, Elias Dahdouh:** writing – review and editing.

## Ethics Statement

In this study, bacterial isolates that were already stored in the relative center's bacterial collection were reused. No samples were specifically acquired for this study.

## Consent

No patient data nor data regarding the clinical sample from which the isolates were acquired were accessed or included. Data were not communicated to healthcare professionals in a way that could affect the patients' care, and only bulk analyses were presented, with no way of tracking each isolate. Consent for publication is not applicable.

## Conflicts of Interest

The authors declare no conflicts of interest.

## Supporting information


**Table S1:** Sequences, concentrations, annealing temperatures, and expected amplicon sizes for the primers used in this study.

## Data Availability

All data and methodology used are present in the manuscript and its Supporting Documents. The raw sequences obtained from amplicon sequencing were deposited in the European Nucleotide Archive (ENA) under Project Number PRJEB83961 (https://www.ebi.ac.uk/ena/browser/view/PRJEB83961, last accessed on 29/10/2025).
